# Independent Determinants of Appetite Impairment among Patients with Stage 3 or Higher Chronic Kidney Disease: A Prospective Study

**DOI:** 10.3390/nu13082863

**Published:** 2021-08-20

**Authors:** Chih-Chien Sung, Min-Tser Liao, Chia-Ter Chao

**Affiliations:** 1 Division of Nephrology, Department of Medicine, Tri-Service General Hospital, National Defense Medical Center, Taipei 11490, Taiwan; sungchihchien@gmail.com; 2 Department of Pediatrics, Taoyuan Armed Forces General Hospital, Taoyuan County 325, Taiwan; 3 Department of Pediatrics, Taoyuan Armed Forces General Hospital Hsinchu Branch, Hsinchu City 300, Taiwan; 4 Department of Pediatrics, Tri-Service General Hospital, National Defense Medical Center, Taipei 11490, Taiwan; 5 Division of Nephrology, Department of Internal Medicine, National Taiwan University Hospital BeiHu Branch, Taipei 10845, Taiwan; 6 Division of Nephrology, Department of Internal Medicine, National Taiwan University College of Medicine, Taipei 100233, Taiwan; 7 Graduate Institute of Toxicology, National Taiwan University College of Medicine, Taipei 100, Taiwan; 8 Division of Nephrology, Department of Internal Medicine, National Taiwan University Hospital, Taipei 100225, Taiwan

**Keywords:** anorexia, appetite, chronic kidney disease, disability, end-stage renal disease, frail phenotype, frailty, functional impairment, malnutrition, protein-energy wasting

## Abstract

Protein-energy wasting (PEW) is an important complication resulting from chronic kidney disease (CKD). Appetite impairment contributes significantly to PEW in these patients, but risk factors associated with having appetite impairment in patients with CKD remain elusive. Patients with an estimated glomerular filtration rate <60 mL/min/1.73 m^2^ for ≥2 times at least three months apart were prospectively enrolled during 2017, with their demographic features, comorbidities, anthropometric parameters, physical and performance indices, functional status, frailty, sensory organ integrity, and laboratory data collected. Their appetite status was measured using the Council on Nutrition Appetite Questionnaire (CNAQ). We examined independent determinants of appetite impairment in these CKD patients using multiple regression analyses. Among 78 patients with CKD, 42.3% had CNAQ-identified impaired appetite. Those with an impaired appetite also had poorer physical performance, a higher degree of functional impairment, higher frail severities, lower serum sodium levels, less intact oral cavity, and a trend toward having less intact nasal structures than those without. Multiple regression analyses revealed that a higher frail severity, in the forms of increasing Study of Osteoporotic Fractures (SOF) scores (odds ratio (OR), 2.74; 95% confidence interval (CI), 1.15–6.57) and a less intact nasal structure (OR, 0.96; 95% CI, 0.92–0.995) were associated with a higher probability of having an impaired appetite, while higher serum sodium (OR, 0.76; 95% CI, 0.6–0.97) correlated with a lower probability. Based on our findings, in patients with CKD, the severity of frailty, serum sodium, and nasal structural integrity might modify appetite status. Therapies targeting these factors might be beneficial for appetite restoration in patients with CKD.

## 1. Introduction

Chronic kidney disease (CKD) is recognized based on abnormalities involving renal structure and/or function persisting for more than three months, and has gradually become a global public health concern since nearly two decades ago. Although a precise estimation of CKD prevalence is difficult due to its asymptomatic nature during its early stage, more than 10% of the population worldwide have CKD [1], and the number may increase even more rapidly with population aging. This trend is alarming, since CKD worsens patients’ prognosis through increasing their risk of mortality. From this perspective, earlier detection of CKD and its complications followed by optimizing dietary and pharmacologic treatment strategies become an important care approach for this ever-expanding population.

A major CKD complication is protein-energy wasting (PEW), which describes the co-existence of depleting protein building blocks and energy stores accompanied by chronic inflammation. Approximately 10–50% of patients with CKD are found to have PEW, and PEW prevalence tends to increase, paralleling the severity of renal dysfunction [2]. The presence of PEW is closely linked to the subsequent development of lean mass loss, sarcopenia, and physical frailty, as well as excessive mortality and healthcare consumption [3]. A multitude of pathogenic factors act in concert to initiate and perpetuate PEW in patients with CKD; consensus from the International Society of Renal Nutrition and Metabolism has identified the driving forces responsible for PEW development, including decreased protein/energy intake, hypermetabolism and anabolic resistance associated with CKD, metabolic acidosis, sequels of comorbidity, and dialysis-related factors [4]. Among these factors, inadequate nutrient consumption seems to be more influential than the others. Patients with non-dialysis CKD often voluntarily limit their protein intake to retard renal function deterioration. Furthermore, existing studies conclude that dietary intake among patients with CKD falls short of the amount recommended by guidelines [5]. Anorexia, or poor appetite, undoubtedly plays an instrumental role in the pathogenesis of this phenomenon.

Anorexia occurs in more than one-third of patients with end-stage renal disease (ESRD) [6] and is responsible for their reduced macronutrient intake. Accumulating uremic toxins, alteration in gut microbiota, metabolic derangement, and various psychological factors all contribute to the development and persistence of anorexia and poor appetite in patients with renal dysfunction [7]. Appetite regulators such as gastrointestinal hormones (cholecystokinin, ghrelin, and others), hypothalamic function, and the integrity of digestive organs are all compromised in patients with CKD [4]. Progressive renal function decline is also accompanied by the development of aggravating anorexia and appetite deterioration. Despite these understandings about CKD (mostly ESRD)-associated appetite impairment, few studies examine factors that further modulate appetite status in those who already have CKD, especially non-dialysis ones. In this study, we aimed to analyze the independent factors, including demographics, comorbidities, physical parameters, functional and frailty status, sensory function, and laboratory profiles, that were capable of influencing the probability of appetite impairment among patients with CKD. We investigated this hypothesis using a standardized appetite gauging questionnaire.

## 2. Methods

The protocol of this study was approved by the Institutional Review Board of the National Taiwan University Hospital (No. 201710041RIND). All participants provided written informed consent. The conductance of this study adhered to the Declaration of Helsinki.

The recruitment procedure has been detailed previously [8]. In brief, we prospectively enrolled participants with CKD from the nephrology and general medicine clinics between 2017 and 2018. Eligible patients included those with an estimated glomerular filtration rate (eGFR) <60 mL/min/1.73 m^2^ for ≥2 times at out-patient settings at least three months apart, with or without hematuria or proteinuria, according to the Kidney Disease Improving Global Outcomes (KDIGO) recommendation [9]. Exclusion criteria consisted of pediatric cases, those who refused to participate, and those unable to provide written informed consent. Upon enrolment, participants received an in-person interview regarding their demographic features, religious beliefs, and relevant comorbidities. This was followed by a dedicated physical examination, during which anthropometric parameters (body height, weight, body mass index (BMI), and waist circumference) and physical indicators (blood pressure (BP) and heart rate (HR)) were recorded prior to their regular clinic visits by pre-trained nursing staff. The physical indicators were measured at rest in a sitting position. In addition, participants were instructed to complete physical performance tests for upper and lower limbs, including the dominant hand grip strength measurement, the time up-and-go test (TUG), the timed chair stand test (TCS), and the gait speed test (GS) [8,10]. For grip strength, we used a digital dynamometer (T.K.K.5401; Takei Scientific Instruments, Niigata, Japan) to obtain the highest readings after repeated measurement. For TUG, we asked participants to stand up from the sitting position and ambulate unassisted for 3 m. For TCS, we asked participants to rise from sitting position to standing repeatedly, followed by measuring the required duration. The results from these tests were obtained after repeating the maneuvers at least twice and calculating the averages. 

These participants were further inquired about their functional status and received frailty screening. Functional status was gauged using two indices, the Eastern Cooperative Oncology Group (ECOG) score and the Karnofsky performance scale. Scores from the former index range between 0 and 5, with higher scores indicating a poorer performance. Scores from the latter index range between 0 and 100, with higher scores indicating better performance. Frailty status was evaluated using the FRAIL scale and the Study of Osteoporotic Fractures (SOF) scale. The former scale operationalizes the concept of frailty using five dimensions, namely, Fatigue, Resistance, Ambulation, Illness, and Loss of weight [11], with a score ranging between 0 and 5, hence the acronym FRAIL. The latter scale characterizes frailty based on three features, weight loss, lower limb weakness, and fatigability, with a score ranging between 0 and 3 [12]. Both the FRAIL and SOF scales have been tested and validated in local older adults [13] and in patients with CKD [14], with good inter-rater reliability and outcome correlations. 

Therefore, in this study, we asked our participants to report whether they had anatomical or functional impairment regarding their smell and taste perceptions, based on a visual analogue scale (VAS) (score range, 0–100). Higher VAS scores indicate better ratings of the specified structure or function. Self-report questions for assessing nasal structure integrity were administered in the following format: “Do you rate your nasal cavity/structure as normal?”.

After completing the interview, self-report questionnaires, and physical tests, all participants received blood tests to determine their hemogram and serum biochemical profiles, including nutritional features, electrolyte panels, and renal function tests. We calculated eGFR based on the Modification of Diet in Renal Disease (MDRD) formula [15].

In this study, appetite status was assessed using the Council on Nutrition Appetite Questionnaire (CNAQ). The CNAQ is an eight-item questionnaire originally designed to evaluate recipients’ appetite and their eating behavior [16]. Each item is rated using a 5-point Likert type scale, with lower scores indicating poorer functions involving that item (score range, 8–40). The CNAQ has been translated into Chinese, with credible results obtained in the general population [17]. We defined patients with a CNAQ score 28 or lower as having an impaired appetite [16].

We described continuous and categorical variables using means with standard deviations, as well as minimal and maximal values in parentheses and numbers with percentages. They were compared between groups using Student’s *t*-tests (for continuous variables) and chi-square tests (for categorical variables), respectively. We first examined the distribution of the CNAQ scores among our CKD participants graphically. This was followed by categorizing participants into those with and without a CNAQ-defined impaired appetite. Univariate analyses were conducted by comparing clinical features, including demographic features, religious beliefs, comorbidities, physical parameters, performance status, sensory functions, frailty test results, and laboratory profiles between participants with and without an impaired appetite. Multiple logistic regression analyses were subsequently performed with an impaired appetite as the dependent variable, incorporating variables with a *p*-value < 0.1 during univariate analyses. We constructed two regression models: Model 1 included demographic features, comorbidities, physical parameters, performance status, frailty test results, and laboratory profiles in the analysis, while model 2 additionally included the sensory function results. SPSS version 19 was used in all analyses, and a two-sided *p*-value < 0.05 was considered statistically significant.

## 3. Results

During the study period, a total of 78 participants with stage 3 or higher CKD were prospectively enrolled, after excluding two patients that could not comply with the study protocol. Only one patient had dialysis-dependent CKD. Their mean age was 72.0 ± 11.7 years, with 55 (70.5%) males. The most common comorbidity among the enrolled participants with CKD was hypertension (*n* = 63; 80.8%), followed by diabetes mellitus (*n* = 54; 69.2%), prior cerebrovascular events (*n* = 11; 14.1%), and malignancy (*n* = 10; 12.8%). The mean systolic and diastolic BP, HR, and BMI were 129.0 ± 15.9 mmHg, 77.1 ± 13.1 mmHg, 75.3 ± 10.1 per min, and 25.3 ± 3.7 kg/m^2^, respectively. Their physical performance was mildly reduced, with mean TCS, TUG, and GS of 12.9 ± 5.5 s, 4.1 ± 3.2 s, and 0.9 ± 0.4 m/s, respectively. Participants with CKD were not anemic (hemoglobin, 7.4 ± 1.1 mmol/L) and their serum albumin levels were 41 ± 3 g/L. Their mean eGFR levels were 31.0 ± 15.1 mL/min/1.73 m^2^.

**Figure 1:**
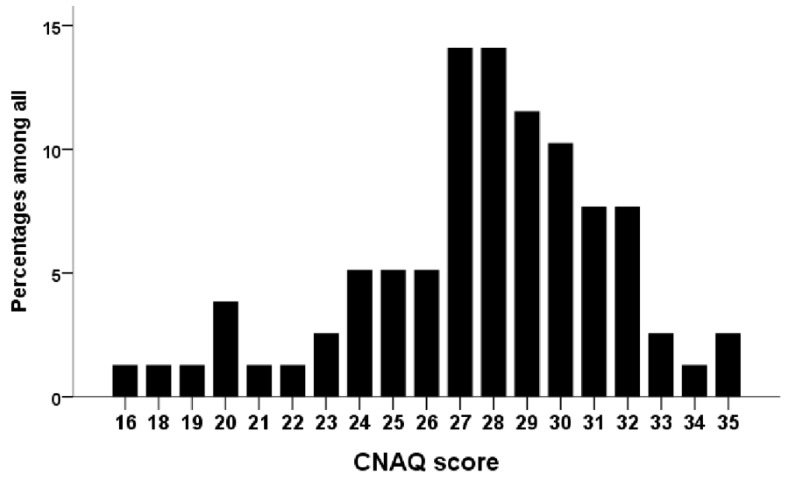
The distribution of the CNAQ scores in the current cohort. CNAQ, Council on Nutrition Appetite Questionnaire.

The distribution of the CNAQ scores is shown in Figure 1. Among all patients with CKD, 42.3% had an impaired appetite. There were no significant differences between CKD patients with and without an impaired appetite regarding age, comorbidities, anthropometric parameters, and most laboratory parameters (Table 1). Those with an impaired appetite were found more likely to be female (*p* = 0.03), had significantly poorer physical performance, including lower grip strength (*p* = 0.04) and longer TCS (*p* = 0.04), had a more severely impaired functional status (*p* = 0.02 and 0.04 for the ECOG and Karnofsky scales, respectively), had higher frail severities, and had lower serum sodium levels (*p* = 0.02) compared to those without (Table 1). A trend of lower hemoglobin (*p* = 0.06) and longer TUG (*p* = 0.06) was observed n CKD patients with an impaired appetite than in those without. Interestingly, CKD patients with an impaired appetite had less self-reported intactness involving their taste function (*p* = 0.04) and oral cavity structure (*p* = 0.02) and had a trend of less intact nasal structure (*p* = 0.07) than those without (Table 1).

We subsequently examined the independent determinants of having an impaired appetite among these with CKD using multiple regression analyses. In model 1, we accounted for variables with a univariate *p*-value < 0.1 (gender, grip strength, TCS, TUG, Karnofsky scale, FRAIL and SOF scores, hemoglobin, and sodium), and discovered that higher SOF scores or an increased frail severity was significantly associated with an elevated probability of having an impaired appetite (odds ratio (OR), 3.42; 95% confidence interval (CI), 1.54–7.6), while hemoglobin levels exhibited a trend of an inverse relationship with the probability of having an impaired appetite (OR, 0.76; 95% CI, 0.57–1.01) (Table 2). In model 2, we additionally incorporated the sensory function results. The results showed that frail severities still positively correlated with the probability of having an impaired appetite (OR, 2.74; 95% CI, 1.15–6.57); however, higher serum sodium levels were associated with a lower probability (OR, 0.76; 95% CI, 0.6–0.97), while having a less intact nasal structure predicted a higher probability (OR, 0.96 per VAS score; 95% CI, 0.92–0.995) of an impaired appetite in patients with CKD (Table 2). Finally, we also evaluated whether BMI and grip strength were associated with appetite impairment in these patients. After incorporating the demographic variables (age and gender), neither BMI (OR, 1.02; *p* = 0.75) nor grip strength (OR, 0.97; *p* = 0.35) were significant determinants of appetite impairment.

## 4. Discussion

In this study, we prospectively recruited a cohort of patients with CKD, among whom the independent determinants of developing appetite impairment were analyzed. After accounting for an exhaustive set of variables, we found that frailty, serum sodium levels, and self-reported less intact nasal function modulated the probability of having appetite impairment. These findings are expected to facilitate a deeper understanding of the origin of appetite status variation and impairment among patients with CKD, and potentially guide the selection of at-risk patients for pre-emptive nutritional care.

Among our participants, 42.3% had an impaired appetite, a number comparable to that in patients with ESRD under chronic dialysis [6]. Relatively few reports address the prevalence of an impaired appetite in patients with non-dialysis CKD, and the existing ones mostly used quality of life measures to qualitatively describe patients’ feelings. For example, Grove et al. found that only 3.9% of patients with pre-dialysis CKD reported having an impaired appetite, using a simple self-report outcome measure [18]. On the contrary, another group identified a nearly 50% prevalence of having an impaired appetite among patients with non-dialysis stage 5 CKD [19]. Our findings of a higher-than-expected prevalence of impaired appetite may have several reasons. It is likely that their advanced age and multimorbidity (Table 1) partially account for this relatively increased prevalence of appetite impairment. Large-scale studies have shown that higher age and female sex are features associated with a lower dietary intake and a higher probability of carrying malnutrition [20], and their appetite may be further suppressed by renal dysfunction. In addition, the higher sensitivity of CNAQ for detecting appetite changes compared to that of other appetite assessment tools could also be responsible [21]. The CNAQ carries the advantages of being a brief yet concise single-domain instrument for appetite assessment, compared to the mini-nutritional assessment (MNA) tool and its short form (MNA-SF), both of which are lengthy and laborious for providers to administer [22].

The association between having more severe frailty and a higher probability of having appetite impairment is reasonable. Older adults with chronic illnesses and subjective complaints such as fatigue, a core component of frailty, are found to exhibit worse feeding behavior and have poorer meal quality and lower frequencies of food intake than those without such complaints. Physical restraints, including difficulty in ambulation and the associated sarcopenia, also correlate closely with having an impaired appetite, especially in older adults. The presence of frailty is frequently accompanied by a higher probability of depression in patients with CKD [23], and depression is associated with centrally-mediated appetite impairment. Finally, frailty predisposes patients with CKD to a greater degree of healthcare resource utilization, and frail individuals tend to be offered inappropriate interventions without outcome benefits. These unnecessary procedures inevitably shorten their duration of meal intake and negatively influence their appetite. Based on these data, frailty may be closely linked to the development and aggravation of appetite impairment, particularly among those whose appetite is already compromised, such as patients with CKD.

It is interesting that neither BMI or abdominal circumference differed between those with and without appetite impairment. Several reasons could be attributable, as provided below; first, the case number of each group was rather modest, and thereby the statistical efficiency for detecting differences could not be reached. Second, there was a possibility that patients with appetite impairment had a higher prevalence of sarcopenic obesity, which led to the manifestations of similar BMI and waist circumferences to those without appetite impairment, but poorer muscle strength.

We also discovered that lower serum sodium levels are associated with a higher probability of having appetite impairment in patients with CKD (Table 2). Prior reports have suggested that dietary sodium intake might interfere with taste perception and potentially modulate appetite in patients with CKD, independent of demographic variables [24]. Sodium deficiency also induces neuropsychological alterations; inadequate body sodium storage affects gustatory function, and various psychological aspects, presenting as concentration difficulty, fatigue, and anhedonia, resulting in appetite impairment [25]. In addition, lower serum sodium levels are frequently associated with an excessive anti-diuretic hormone (ADH) release. Changes in the amount of secreted ADH can be indirectly modulated by gut hormones and microbiota composition [26], all of which serve as potential regulators of one’s appetite. ADH has also been shown to increase blood glucose levels and suppress lipolysis [27], while glycemic variations may influence several appetite-regulating hormones such as ghrelin and leptin. Importantly, ADH may potentiate the release of stress hormones such as adrenocorticotropic hormone and cortisol [27]. The resultant inflammation status attenuates ghrelin secretion and leads to appetite impairment [28], especially in patients with CKD. It is then plausible that serum sodium levels may play an under-recognized role in modifying the appetite of patients with CKD. 

Sensory perceptions, especially smell and taste intactness, impact heavily on the choices of food preferences and appetite [29]. The finding that subjective nasal complaints, but not oral/taste complaints, in patients with CKD correlate with a higher probability of appetite impairment (Table 2) also warrants attention. Prior studies in older adults have mostly demonstrated that olfactory dysfunction fails to exhibit an association with appetite changes [30]. Researchers frequently use objective olfactory strip-based toolkits for assessment, but very few attempt to characterize whether enrollees have structural lesions in olfactory organs such as their noses. Our findings thus support the possibility that the absence of a connection between olfactory integrity and appetite or nutritional status may be explained by the failure to consider the structural integrity of noses. More studies are needed to confirm our findings.

Vital clinical implications can be derived from our findings. Strategies aiming to ameliorate frailty, including nutritional care, rehabilitation programs, and exercise sessions, may be harnessed to reduce the probability of developing appetite impairment among patients with CKD [31,32,33]. Several risk factors for frailty have been uncovered by our group before, serving as a guide for potential therapeutic targets [34,35,36]. In addition, other geriatric phenotypic clusters, such as multimorbidity and cognitive impairment, may also influence the association between frailty and appetite impairment in this study [37]. However, frailty-targeting treatments may not necessarily benefit those with CKD regarding appetite improvement; these patients are similarly at risk of developing cardiovascular events, and therefore a multipronged care strategy may be better suited for optimizing outcomes [38]. Those with abnormal serum sodium, especially those with lower levels, as well as those with nasal lesions, may be subgroups of CKD patients that are particularly at risk, and will likely benefit more from such interventions. These patients may be advised to receive screening for appetite change regularly. 

Our study has its advantages and drawbacks. The focus of this study, to determine the independent determinants of an impaired appetite in patients with CKD, fills the research gap and enriches the existing literature by providing evidence on potential appetite modifiers in patients with CKD. We compiled a comprehensive set of variables for selecting the potential determinants of appetite impairment, defined in a standardized way, and some of the included factors (e.g., frailty, functional status, and sensory function) have not been examined before for such an appetite-influencing effect. However, several limitations should be kept in mind when interpreting our results. First, the findings from our study cannot affirm whether a causal relationship exists between the identified factors and the probability of having an impaired appetite in CKD patients. Our sample size was modest, but the large number of variables analyzed most likely negates the influence posed by statistical inefficiency. The assessment of appetite, although conducted using a structuralized instrument, remained subjective in nature and might not always be compatible with results using objective nutritional assessment methods. In addition, depression was not measured in this study. Finally, patients in our cohort mostly had stages 3 and 4 CKD, and extrapolating our results to those with dialysis-dependent CKD should be conducted very carefully. Nonetheless, a replicative study is still needed to verify our findings.

## 5. Conclusions

In conclusion, we revealed several important yet under-recognized factors that independently influence the probability of having appetite impairment in patients with CKD. The severity of frailty, serum sodium levels, and the presence of nasal structural lesions all play a role in modifying appetite status. Therapies targeting these factors might become potential approaches for appetite restoration in these patients.

## Figures and Tables

**Table 1:** Characteristics of patients with chronic kidney disease with and without an impaired appetite.

	With Impaired Appetite (*n* = 33)	Without Impaired Appetite (*n* = 45)	*p*-Value
Baseline features			
Age (years)	71.8 ± 14.4 (34.1, 92.8)	72.1 ± 9.3 (48.8, 87.9)	0.93
Male sex (%)	19 (58)	36 (80)	0.03
Religion			0.28
None (%)	8 (24)	6 (13)	
Buddhism (%)	14 (42)	24 (53)	
Taoism (%)	8 (24)	14 (31)	
Christianity (%)	3 (9)	1 (2)	
Concurrent illnesses			
Coronary artery disease (%)	3 (9)	2 (4)	0.41
Hypertension (%)	28 (85)	35 (78)	0.44
Prior MI (%)	2 (6)	2 (4)	0.75
Heart failure (%)	1 (3)	1 (2)	0.83
Atrial fibrillation (%)	2 (6)	1 (2)	0.39
Prior cerebrovascular events (%)	3 (9)	8 (18)	0.28
Diabetes mellitus (%)	19 (58)	35 (78)	0.71
Cirrhosis (%)	1 (3)	0 (0)	0.25
COPD (%)	0 (0)	1 (2)	0.40
Malignancy (%)	6 (18)	4 (9)	0.23
Autoimmune diseases (%)	2 (6)	3 (7)	0.92
Gastric/duodenal ulcer (%)	2 (6)	6 (13)	0.30
Dementia (%)	1 (3)	0 (0)	0.25
Anthropometric parameters and physical status			
Systolic BP (mmHg)	129.7 ± 14.9 (100, 158)	128.5 ± 16.8 (97, 174)	0.76
Diastolic BP (mmHg)	68.9 ± 12.9 (42, 92)	69.3 ± 13.4 (43, 116)	0.39
Heart rate (per min)	75.7 ± 11.1 (58, 99)	75.0 ± 9.5 (56, 95)	0.76
Weight (kg)	66.5 ± 13.6 (41, 96)	68.0 ± 11.0 (48, 92)	0.60
Height (cm)	161.0 ± 9.1 (145, 178)	164.0 ± 6.7 (149, 179)	0.10
Body mass index (kg/m^2^)	25.5 ± 4.3 (18.2, 37)	25.2 ± 3.2 (18.8, 32.6)	0.68
Waist circumference (cm)	91.8 ± 12.7 (63, 115)	91.2 ± 9.6 (66, 110)	0.83
Grip strength (kg)	21.3 ± 9.4 (8.1, 45.5)	25.6 ± 8.6 (10.7, 42.6)	0.04
Time up-and-go (s)	4.9 ± 3.9 (1.5, 16)	3.5 ± 2.6 (1.3, 16.6)	0.06
Timed chair stand (s)	14.4 ± 6.2 (8.3, 34.3)	11.8 ± 4.6 (5.4, 27.1)	0.04
Gait speed (m/s)	0.8 ± 0.4 (0.21, 1.58)	1.0 ± 0.4 (0.16, 1.83)	0.11
Functional status and frailty			
ECOG	0.76 ± 0.83 (0, 3)	0.36 ± 0.65 (0, 2)	0.02
Karnofsky performance scores	89.4 ± 13.7 (50, 100)	95.1 ± 9.9 (60, 100)	0.04
SOF scores	0.88 ± 0.89 (0, 3)	0.33 ± 0.48 (0, 1)	<0.01
FRAIL scores	0.91 ± 1.18 (0, 4)	0.44 ± 0.62 (0, 2)	0.03
Sensory function (self-rated)			
Any nasal complaint (0–100)	74.8 ± 17.9 (20, 100)	81.8 ± 14.9 (50, 100)	0.07
Any taste complaint (0–100)	75.6 ± 16.9 (30, 100)	82.8 ± 14.0 (30, 100)	0.04
Any oral cavity complaint (0–100)	67.6 ± 19.8 (20, 100)	77.9 ± 19.2 (30, 100)	0.02
Laboratory test results			
Nutrition			
Albumin (g/L)	41 ± 3 (33, 48)	42 ± 3 (34, 49)	0.18
Fasting glucose (mmol/L)	6.6 ± 2.0 (4.3, 12.5)	6.5 ± 1.9 (4.1, 14.9)	0.72
Total cholesterol (mmol/L)	4.4 ± 1.4 (2.5, 9.4)	4.4 ± 0.8 (2.1, 6.1)	0.99
Triglyceride (mmol/L)	1.6 ± 0.8 (0.6, 4.0)	1.5 ± 0.8 (0.6, 4.1)	0.81
Rheologic			
Leukocyte (K/μL)	6.6 ± 2.0 (1.5, 11.7)	6.4 ± 1.6 (3.2, 9.6)	0.61
Hemoglobin (mmol/L)	7.1 ± 1.1 (5.1, 10.2)	7.6 ± 1.1 (4.8, 10.1)	0.06
Platelet (K/μL)	214.4 ± 69.8 (29, 385)	213.9 ± 67.6 (78, 405)	0.98
Electrolyte			
Sodium (meq/L)	136.9 ± 2.9 (128, 141)	138.4 ± 2.4 (133, 145)	0.02
Potassium (meq/L)	4.6 ± 0.4 (3.9, 5.5)	4.5 ± 0.5 (3.1, 5.5)	0.53
Calcium (mmol/L)	2.3 ± 0.1 (1.9, 2.6)	2.3 ± 0.1 (1.8, 2.6)	0.72
Phosphorus (mmol/L)	1.2 ± 0.2 (0.9, 1.7)	1.2 ± 0.3 (0.8, 1.8)	0.99
Renal function			
Urea nitrogen (mmol/L)	15.4 ± 6.6 (6.5, 36.4)	13.9 ± 5.4 (5.3, 34.3)	0.25
Creatinine (μmol/L)	300.6 ± 380.2 (88.4, 1830)	282.9 ± 344.8 (106.1, 2299)	0.82
eGFR (mL/min/1.73 m^2^)	31.7 ± 16.1 (3, 58.5)	30.4 ± 14.5 (2, 63.1)	0.71

Values are expressed in means ± standard deviation with minimal and maximal values in parentheses, or numbers with percentages in parentheses. BP, blood pressure; COPD, chronic obstructive pulmonary disease; ECOG, Eastern Cooperative Oncology Group; eGFR, estimated glomerular filtration rate; MI, myocardial infarction; SOF, Study of Osteoporotic Fractures.

**Table 2:** Regression analysis with having an impaired appetite as the dependent variable (*n* = 78).

Variables	OR	95% CI	*p*-Value
Model 1 *			
Hemoglobin	0.76	0.57–1.01	0.06
SOF score	3.42	1.54–7.60	<0.01
Model 2: Model 1 + Sensory function variables			
Sodium (meq/L)	0.76	0.60–0.97	0.03
SOF score	2.74	1.15–6.57	0.02
Any nasal complaint	0.96	0.92–0.995	0.03

Model 1: Including gender, grip strength, TCS, TUG, Karnofsky scale, FRAIL and SOF scores, hemoglobin, and sodium. CI, confidence interval; OR, odds ratio; SOF, Study of Osteoporotic Fractures. * Incorporated variables with differences in the univariate analysis.

## Data Availability

Data will be available upon reasonable request to the corresponding author(s).
